# Increase of catastrophic and impoverishing health expenditures in Mexico associated to policy changes and the COVID-19 pandemic

**DOI:** 10.7189/jogh.13.06044

**Published:** 2023-10-27

**Authors:** Edson Serván-Mori, Octavio Gómez-Dantés, David Contreras, Laura Flamand, Diego Cerecero-García, Héctor Arreola-Ornelas, Felicia M Knaul

**Affiliations:** 1 Center for Health Systems Research, National Institute of Public Health, Cuernavaca, Morelos, Mexico; 2 Institute for Obesity Research, Tecnologico de Monterrey, Mexico; 3 School Government and Public Transformation, Tecnologico de Monterrey, Mexico; 4 Center for International Studies, El Colegio de Mexico, Mexico City, Mexico; 5 Public Health Policy Evaluation Unit, Imperial College London, London, United Kingdom; 6 Mexican Health Foundation (FUNSALUD), Mexico; 7 Tomatelo a Pecho, A.C., Mexico; 8 The University of Miami Institute for Advanced Study of the Americas, USA; 9 Miller School of Medicine, University of Miami, USA

## Abstract

**Background:**

In 2003, the Mexican Congress approved a major reform to provide health care services to the poor population through the public insurance scheme *Seguro Popular*. This program was dismantled in 2019 as part of a set of health system reforms and substituted with the Health Institute for Welfare (INSABI). These changes were implemented during the initial phases of the coronavirus (COVID-19) pandemic. We aimed to examine the impact of these reforms and the COVID-19 pandemic on financial risk protection in Mexico between 2018 and 2020.

**Methods:**

We performed a population-based analysis using cross-sectional data from the 2018 and 2020 rounds of the National Household Income and Expenditures Survey. We used a pooled fixed-effects multivariable two-stage probit model to determine the likelihood of catastrophic health expenditure (CHE), impoverishing health expenditure (IHE), and excessive health expenditure (EHE) among Mexican households. We also mapped the quintiles of changes in EHE in households without health insurance by state.

**Results:**

The percentage of households without health insurance almost doubled from 8.8% (three million households) in 2018 to 16.5% (5.8 million households) in 2020. We also found large increases in the proportion of households incurring in CHE (18.4%; 95% confidence interval (CI) = 6.1, 30.7) and EHE (18.7%; 95% CI = 7.9, 29.5). Significant increases in CHE, IHE, and EHE were only observed among households without health insurance (CHE: 90.7%; 95% CI = 31.6, 149.7, EHE: 73.5%; 95% CI = 25.3, 121.8). Virtually all Mexican states (n/N = 31/32) registered an increase in EHE among households without health insurance. This increase has a systematic territorial component affecting mostly central and southern states (range = -1.0% to 194.4%).

**Conclusions:**

The discontinuation of the *Seguro Popular* Program and its substitution with INSABI during the first stages of the COVID-19 pandemic reduced the levels of health care coverage in Mexico. This reduction and the pandemic increased out-of-pocket expenditure in health and the portion of CHE and EHE in the 2018-2020 period. The effect was higher in households without health insurance and households in central and southern states of the country. Further studies are needed to determine the specific effect both of recent policy changes and of the COVID-19 pandemic on the levels of financial protection in health in Mexico.

Households and individuals can be driven to poverty by catastrophic health expenditures or those that exceed a family’s financial capacity [1-6]. Despite global efforts, 100 million households worldwide still fall into extreme poverty each year due to out-of-pocket health expenses [7].

A key factor behind the high prevalence of catastrophic spending is the exclusion of population groups from financial risk protection mechanisms [8]. This shortcoming is common in fragmented systems, characterised by the coexistence of several health care delivery subsystems to provide services to different population groups, financed by various funding pools, and run by specific rules for accessing financial resources and health benefits [9]. Generally, subsystems that offer health care to the poor (which tend to be under the control of the Ministry of Health) are inadequately financed and consequently tend to offer only basic, low-quality services on an assistential basis [10]. Their users usually have to pay out-of-pocket for many health inputs (diagnostic procedures, medicines, and other materials) and interventions, even within public facilities, which produces catastrophic and impoverishing health expenditures [11-14], and are thus frequently forced to migrate to the private sector to access more responsive or complex services not available at public facilities [10]. This generates out-of-pocket expenditures that also produce severe financial shocks for families [14].

Financial protection in health is a core element of universal health coverage, achieved when the use of health care services does not expose households to financial hardship or threaten living standards [15]. Public, prepayment schemes, and pooling of resources to avoid out-of-pocket payments are needed to achieve this type of protection [16].

In 2003, the Mexican Congress approved a major reform to provide health care services to the population without social security through the public insurance scheme *Seguro Popular* [17], which by 2018 guaranteed access to 294 essential services and 66 high-cost interventions with 53.5 million enrolled beneficiaries [18]. *Seguro Popular* had positive impacts, including the expansion of public investment in health, an increase in health care coverage, and a reduction of catastrophic and impoverishing health expenditures.[19]

Despite the documented relative successes of *Seguro Popular*, the federal administration, headed by President Andrés Manuel López Obrador (2018-2024), decided to dismantle it and replace it with the Health Institute for Welfare (*Instituto de Salud para el Bienestar* (*INSABI*)). The dismantling process began in January of 2020 and coincided with the onset of the coronavirus (COVID-19) pandemic. By 2021, *INSABI* had negotiated the provision of health care services to the population without social security in 26 of the 32 federal states [20,21]. The six remaining states decided to provide services to their population without social security following not federal but local rules, although the financing remained mostly federal. *INSABI* created significant uncertainty for patients, health workers, and state governments. For example, its bylaws partially regulating services, finance, and labour conditions were published more than a year after the new institute began operating [21]. In April 2022, the federal government announced that a pre-existing federal program, *IMSS-Bienestar*, would provide health services for the uninsured population instead of *INSABI* [22].

In this article we first describe the methods used to estimate the changes in health care coverage and financial protection in Mexico between 2018 and 2020 associated to the policy changes described above. We then discuss the possible impacts of the dismantling of *Seguro Popular* using a coverage indicator and three types of financial protection indicators. Finally, in section three we discuss the implications of these results for the Mexican health system and for other low- and middle-income countries. Its main contribution is the use of financial protection methods to evaluate the possible short-run impacts of policy changes in health system performance.

## METHODS

### Design and population study

We performed a population-based analysis using cross-sectional data from the 2018 and 2020 rounds of Mexico’s National Household Income and Expenditures Survey (ENIGH) [23], implemented biennially since 1992. ENIGH collects data on household (unit of observation) income and expenditures and on economic activities developed by household members [24]. We use the most recent years to account for the policy changes described in the introduction. The longer time series has been analysed elsewhere [19,25,26]. The ENIGH survey is administered and managed by the National Institute for Statistics and Geography. It includes a standardised set of income and expense questions and is the most complete source of data of health spending in Mexico. It is a probabilistic, stratified, two-stage, clustered sample that allows for the generation of estimates at the national and state level within urban and rural strata [24].

From August to November 2018 and 2020, trained personnel completed face-to-face surveys of 74 647 and 89 006 households, respectively, in each survey. After excluding 0.7% of households with incomplete data or implausible values in the variables of interest for this study, we obtained a sample of 162 204 households, which represented almost 70 million Mexican households when both rounds are combined (2018: n = 34 471 097, 2020: n = 35 449 992).

### Financial risk protection in health

Financial risk protection in health involves protection against financial shocks associated to the use and payment for health care services [5,27,28]. In our study, we operationalised the level of financial risk protection through household health insurance and consequently stratified households according to four types of health insurance status: no public or private insurance, affiliated to *Seguro Popular* and/or *INSABI*, affiliated to social security, and mixed affiliation (household members having more than one source of health insurance, including private insurance).

### Total household consumption and subsistence expenditure

Following previous studies [19,25,29,30], we first calculated the total expenditure (TE) quarterly consumption for each household *i* (TEi) by adding expenditures on food expenditure and beverages (FEi), transportation and communication, housing and services, personal care, education and health expenditure (HEi), among others. We estimated HEi according to the Classification of Individual Consumption by Purpose (COICOP) 2018 [31], including expenditures for medicines and other health products, outpatient care, hospitalisation, and other services such as laboratory analyses and dental services. We then calculated the level of subsistence expenditure (SE) or poverty line as the average of FEi between 45% and 55% of TE, adjusted for equivalence in consumption (equal to household size β), where β was set at 0.56, as reported elsewhere [8]. We then calculated the household capacity to pay (CTPi) equal to TEi - FEi (when SE>FEi) and equal to TEi − SE (when SE≤FEi).

Expenditure figures are those incurred during the quarter prior to the survey and are expressed in international purchasing power parity dollar (PPP) constant dollars of 2013. We complemented the classification of subsistence expenditure with expenditure on food outside the home due to its importance in Mexican households [32]. Similarly, we also included both the monetary and non-monetary components of household spending, the latter being derived from self-consumption or gifts received from other households (at market prices) and institutional contributions, such as government subsidies or private organisations’ transfers [32].

### Outcome variables

Following previous research [19,26], we analysed three binary indicators of financial risk in health care:

1. Catastrophic health-care expenditure (CHEi) was equal to 1, where HEi ≥ 30% of household capacity to pay (CTPi) and otherwise equal to zero [20,33].
2. Impoverishing health expenditures (IHEi), defined as expenditure on health care that results in a household *i* falling below the prevailing poverty line or deepening its impoverishment if it is already poor [34], equal to one if TEi ≥ SE and TEi − HEi < SE, and equal to zero otherwise [33].
3. Excessive health-care expenditure (EHEi) included households with CHE and/or those with impoverishing health expenditures (equal to 1 if TEi ≥ SE and TEi − HEi < SE, and equal to zero otherwise) [25,35,36].

### Covariates

Following previous studies [29,37-43], we included several characteristics in our multivariate analysis at different levels: head of household: age (in years), sex (women = 1, men = 0), schooling level (none, elementary, secondary, high school, and college), employment during the last month (yes = 1, no = 0), and marital status (married/free union, divorced/separated/widowed, and single). Household characteristics: indigenous status (yes = 1, no = 0), composition (unipersonal, nuclear, extended, or composited), number of equivalent adults [44], the proportion of family members aged 0-5 or ≥65 years and with a disability, a factorial asset and housing material standardised index as a measure of socioeconomic status [45], where the higher values indicate a greater number of assets and better housing conditions, and participation in any government conditional/non-conditional transfers program. Area of residence: rurality/urbanity (urban ≥2500 inhabitants) and a factorial social-deprivation index-based data collected by the 2020 Population Census on municipal access to basic public services, housing conditions and salary [46], where the higher values indicate a greater social municipal development.

### Statistical analysis

We performed all analyses using the stat, version 17.0. (StataCorp LLC, College Station, Texas, USA) considering complex survey design and sampling weights using the *svy* package module. We first report averages or row-percentages (with a 95% confidence interval (CI)) of the characteristics described above by each survey wave, including THE, FE, and CTP, the prevalence of households with HE>0, and the HE and the HE/CTP ratio (in percentage), according to health insurance status and survey year.

To adjust the probabilities of CHE, IHE, and EHE (with a 95% CI), we then considered selection bias in the demand for health services (HE>0), estimating a two-stage probit or Heckprobit model [47]. We adjusted the first stage (the outcome equation for CHE, IHE, and EHE) for all covariates described above and for the interaction between survey year and health insurance, and by state and survey year fixed effects. The second stage (or selection equation in the positive HE) was also adjusted for all control variables mentioned above (except by the household composition, the proportion of family members aged 0-5 or ≥65 years and with a disability) and for state and survey year fixed effects. Following the definition of CHE suggested by the Sustainable Development Goal (SDG) 3.8.2. [48] and previous studies [8,14,19,49], we performed sensitivity analyses of our estimates of change in CHE and EHE prevalence by modifying the cutoff thresholds on the HE/CTP ratio to ≥25% and ≥40% (Appendix 1 in the **Online Supplementary Document**).

Finally, we conducted post-hoc comparisons of quintiles of changes (2018-2020) in adjusted prevalence of EHE by state among households without health insurance. We computed percentage changes between 2018-2020 and 95% CI using a nonlinear combination of post estimated parameters based on the delta method, using the *nlcom* command of the Stata statistical package.

### Role of the funding source

The funder of the study had no role in the study design, data analysis, data interpretation, or writing of the report.

## RESULTS

The percentage of households without health insurance almost doubled from 8.8% (3 million households) in 2019 to 16.5% (5.8 million households) in 2020, while there was also a decrease in the affiliation to *Seguro Popular* and/or *INSABI* (30.8% to 21.7%) and mixed affiliation (20.1% to 14.4%). In contrast, the proportion of households with social security increased from 40.3% to 47.4% (**Table 1**). The drop in health insurance coverage was more pronounced in female-headed households, those with lower schooling, those with lower participation in the labour market, indigenous households, those with lower socioeconomic status, and those living below the poverty line (**Table 1**). Among beneficiaries of any government conditional/non-conditional transfers program, the drop-in health insurance coverage affected those in rural contexts and those with more significant social deprivation (**Table 1**).

**Table 1:** Overall characteristics of analysed households and their distribution according to their health insurance status, Mexico 2018-2020*

	No health insurance		Seguro Popular and/or INSABI†		Social security		Mixture	
**Year‡**	**2018**	**2020**	**2018**	**2020**	**2018**	**2020**	**2018**	**2020**
**Population, weighted n (%)**	3 019 668 (8.8)	5 863 429 (16.5)	10 630 886 (30.8)	7 674 923 (21.7)	13 888 405 (40.3)	16 803 296 (47.4)	6 932 138 (20.1)	5 108 344 (14.4)
**Head of household**								
Average age in years	47.0 (46.4, 47.6)	48.9 (48.5, 49.3)	49.0 (48.6, 49.3)	50.0 (49.6, 50.3)	50.8 (50.4, 51.1)	52.2 (52.0, 52.5)	50.7 (50.3, 51.0)	52.7 (52.3, 53.0)
Male	8.8 (8.5, 9.2)	16.6 (16.1, 17.1)	31.6 (30.7, 32.4)	22.4 (21.8, 23.0)	39.8 (38.9, 40.6)	46.8 (46.0, 47.6)	19.8 (19.3, 20.3)	14.2 (13.8, 14.6)
Female	8.5 (7.9, 9.2)	16.4 (15.7, 17.1)	29.0 (28.0, 30.0)	19.9 (19.2, 20.7)	41.6 (40.4, 42.7)	48.8 (47.8, 49.8)	20.9 (20.0, 21.7)	14.9 (14.2, 15.5)
Schooling								
*None*	8.7 (7.5, 9.8)	22.4 (20.9, 23.8)	59.3 (57.3, 61.3)	41.7 (39.9, 43.6)	15.6 (14.1, 17.0)	21.4 (19.9, 23.0)	16.5 (15.1, 17.8)	14.5 (13.2, 15.7)
*Elementary*	7.9 (7.3, 8.4)	18.2 (17.5, 18.9)	44.4 (43.3, 45.5)	31.9 (30.9, 32.8)	25.4 (24.4, 26.3)	33.7 (32.8, 34.7)	22.4 (21.5, 23.2)	16.2 (15.6, 16.9)
*Secondary*	8.6 (8.0, 9.3)	16.7 (16.0, 17.5)	32.6 (31.5, 33.7)	23.7 (22.9, 24.5)	34.4 (33.3, 35.6)	42.5 (41.4, 43.5)	24.3 (23.4, 25.2)	17.1 (16.4, 17.8)
*High school*	9.7 (8.8, 10.5)	16.7 (15.8, 17.7)	20.5 (19.3, 21.7)	13.6 (12.7, 14.4)	51.0 (49.5, 52.5)	56.1 (54.7, 57.4)	18.8 (17.7, 19.9)	13.6 (12.8, 14.5)
*College*	9.6 (8.8, 10.4)	12.4 (11.6, 13.1)	7.6 (7.0, 8.2)	5.5 (5.0, 6.0)	68.9 (67.8, 70.1)	72.7 (71.7, 73.7)	13.9 (13.0, 14.7)	9.4 (8.8, 10.0)
Unemployed in the last month	6.7 (6.1, 7.3)	13.3 (12.6, 14.0)	24.8 (23.7, 25.9)	16.4 (15.6, 17.1)	48.1 (46.8, 49.4)	55.3 (54.3, 56.3)	20.4 (19.5, 21.4)	15.0 (14.4, 15.7)
Employed in the last month	9.3 (8.9, 9.7)	17.7 (17.2, 18.2)	32.5 (31.7, 33.3)	23.6 (23.0, 24.3)	38.2 (37.4, 38.9)	44.5 (43.7, 45.2)	20.0 (19.5, 20.6)	14.2 (13.8, 14.6)
Marital status								
*Married/free union*	7.0 (6.7, 7.4)	14.7 (14.3, 15.2)	32.4 (31.6, 33.3)	23.4 (22.8, 24.1)	39.3 (38.5, 40.1)	46.6 (45.8, 47.3)	21.2 (20.6, 21.8)	15.3 (14.8, 15.7)
*Divorced/separated/widowed*	10.7 (10.0, 11.4)	18.2 (17.4, 19.0)	28.7 (27.6, 29.7)	19.3 (18.5, 20.1)	41.3 (40.1, 42.5)	48.5 (47.4, 49.5)	19.3 (18.4, 20.2)	14.1 (13.4, 14.7)
*Single*	17.4 (16.0, 18.8)	26.2 (24.6, 27.8)	24.0 (22.4, 25.6)	14.4 (13.2, 15.5)	45.5 (43.5, 47.6)	51.0 (49.2, 52.8)	13.1 (11.8, 14.3)	8.4 (7.5, 9.3)
**Household**								
Non-indigenous	8.9 (8.6, 9.3)	16.3 (15.9, 16.8)	28.3 (27.5, 29.1)	19.4 (18.9, 20.0)	42.6 (41.8, 43.4)	49.8 (49.1, 50.5)	20.2 (19.7, 20.7)	14.4 (14.1, 14.8)
Indigenous	7.0 (6.1, 8.0)	18.8 (17.4, 20.2)	58.4 (56.0, 60.9)	47.3 (45.0, 49.7)	15.3 (13.7, 16.9)	19.6 (18.0, 21.3)	19.2 (17.6, 20.8)	14.2 (13.1, 15.4)
Composition								
*Unipersonal*	22.7 (21.4, 24.0)	31.8 (30.5, 33.1)	28.9 (27.5, 30.3)	17.9 (16.9, 18.8)	45.5 (43.8, 47.1)	48.1 (46.6, 49.5)	3.0 (2.5, 3.4)	2.2 (1.8, 2.7)
*Nuclear*	8.2 (7.7, 8.6)	16.5 (16.0, 17.0)	32.3 (31.5, 33.2)	22.5 (21.9, 23.2)	42.4 (41.5, 43.3)	49.2 (48.4, 50.0)	17.1 (16.6, 17.6)	11.8 (11.4, 12.2)
*Extended*	3.9 (3.4, 4.3)	9.6 (9.0, 10.1)	28.5 (27.4, 29.6)	21.5 (20.6, 22.4)	32.3 (31.1, 33.5)	42.7 (41.7, 43.8)	35.3 (34.2, 36.4)	26.2 (25.4, 27.1)
*Composite*	6.3 (4.1, 8.6)	15.2 (11.6, 18.7)	19.5 (15.3, 23.8)	16.1 (13.1, 19.1)	42.2 (36.9, 47.4)	47.8 (43.2, 52.4)	32.0 (27.0, 36.9)	20.9 (17.6, 24.3)
Average number of equivalent adults	2.1 (2.0, 2.1)	2.2 (2.2, 2.2)	2.6 (2.6, 2.6)	2.6 (2.6, 2.7)	2.4 (2.4, 2.4)	2.4 (2.4, 2.5)	3.1 (3.1, 3.1)	3.1 (3.1, 3.2)
Family members aged 0-5	9.6 (9.2, 10.0)	17.2 (16.8, 17.7)	27.8 (27.0, 28.5)	19.3 (18.8, 19.9)	44.4 (43.5, 45.2)	50.8 (50.0, 51.5)	18.3 (17.7, 18.8)	12.7 (12.3, 13.0)
Without family members aged 0-5	6.5 (6.0, 7.1)	14.2 (13.5, 14.9)	39.3 (38.0, 40.5)	29.2 (28.2, 30.2)	29.1 (28.1, 30.1)	36.5 (35.4, 37.6)	25.1 (24.2, 26.0)	20.1 (19.3, 20.9)
Family members aged ≥65	9.6 (9.2, 10.0)	17.9 (17.4, 18.3)	31.0 (30.2, 31.8)	22.0 (21.4, 22.6)	39.7 (38.9, 40.5)	46.4 (45.6, 47.2)	19.7 (19.2, 20.2)	13.8 (13.4, 14.2)
Without family members aged ≥65	5.9 (5.4, 6.5)	12.6 (12.0, 13.3)	30.3 (29.1, 31.4)	20.7 (19.8, 21.5)	42.3 (41.0, 43.6)	50.3 (49.3, 51.4)	21.5 (20.6, 22.4)	16.4 (15.6, 17.1)
Family members with a disability	9.6 (9.2, 10.0)	17.3 (16.8, 17.7)	29.8 (29.0, 30.6)	20.8 (20.2, 21.4)	41.6 (40.8, 42.5)	48.5 (47.8, 49.3)	19.0 (18.5, 19.5)	13.4 (13.0, 13.8)
Without family members with a disability	5.7 (5.2, 6.3)	13.4 (12.7, 14.1)	34.8 (33.6, 36.0)	25.2 (24.3, 26.1)	35.3 (34.1, 36.5)	42.6 (41.5, 43.7)	24.1 (23.2, 25.1)	18.8 (18.0, 19.6)
SES index (SD)	-21.6 (-28.6, -14.5)	-35.6 (-40.7, -30.6)	-118.4 (-124.4, -112.4)	-103.8 (-109.7, -97.9)	71.5 (69.5, 73.4)	72.5 (70.8, 74.1)	7.0 (3.4, 10.6)	13.9 (10.4, 17.5)
Over SE (or poverty line-PL)	9.0 (8.7, 9.4)	16.2 (15.8, 16.7)	26.7 (25.9, 27.4)	18.5 (18.0, 19.0)	43.8 (43.0, 44.5)	50.7 (50.0, 51.3)	20.5 (20.0, 21.1)	14.6 (14.2, 14.9)
Below SE (or poverty line-PL)	6.2 (5.5, 7.0)	19.5 (18.3, 20.7)	68.5 (66.9, 70.1)	52.3 (50.5, 54.1)	9.1 (8.2, 10.0)	15.3 (14.1, 16.6)	16.1 (15.0, 17.3)	12.9 (11.9, 13.9)
Non-beneficiary of any social program	11.2 (10.7, 11.6)	18.9 (18.4, 19.4)	21.6 (20.9, 22.2)	18.3 (17.8, 18.9)	49.5 (48.7, 50.3)	50.7 (49.9, 51.4)	17.8 (17.3, 18.3)	12.1 (11.7, 12.5)
Beneficiary of any social program	2.6 (2.3, 3.0)	11.0 (10.4, 11.5)	54.6 (53.3, 55.8)	29.5 (28.5, 30.5)	16.9 (16.0, 17.7)	39.7 (38.7, 40.7)	25.9 (25.0, 26.9)	19.9 (19.2, 20.6)
**Area of residence**								
Rural	6.6 (6.1, 7.1)	18.6 (17.8, 19.4)	60.7 (59.2, 62.1)	46.0 (44.7, 47.3)	14.6 (13.6, 15.5)	19.8 (18.7, 20.8)	18.2 (17.4, 18.9)	15.7 (15.0, 16.3)
Urban	9.4 (9.0, 9.8)	16.0 (15.5, 16.5)	21.9 (21.0, 22.7)	15.0 (14.4, 15.6)	48.0 (47.1, 49.0)	55.0 (54.2, 55.8)	20.7 (20.1, 21.3)	14.1 (13.6, 14.5)
Social deprivation index (SD)	-14.4 (-19.0, -9.7)	10.6 (6.5, 14.7)	85.9 (79.8, 92.0)	87.2 (81.3, 93.2)	-52.8 (-55.0, -50.7)	-49.9 (-51.7, -48.1)	-5.2 (-9.0, -1.5)	1.4 (-2.3, 5.0)

SES – socioeconomic status, SE – subsistence expenditure, PL – poverty line, SD – standard deviation, INSABI – Instituto de Salud para el Bienestar

*Data presented as calculated average or percentage (95% CI) unless specified otherwise.

†Refers to any government conditional/non-conditional. In particular, the Conditional-Cash-Transfer Program known as Prospera (formerly Progresa or Oportunidades) (POP program) which was recently cancelled.

‡Data from 2018 and 2020 waves of the National Household Income and Expenditure Survey (ENIGH).

From 2018 to 2020, THE fell by -11.0% (95% CI = -12.6, -9.3), from approximately $PPP3160 to $PPP2800, with a similar trend for FE and and even more pronounced one for CTP (-12.5%; 95% CI = -14.7, -10.3) (**Table 2**). In contrast, household probability of incurring in HE increased by 17.2% (95% CI = 15.5, 18.9). HE (among households which reported HE>0) also decreased by -7.5% (95% CI = -12.7, -2.2), from $PPP180 to $PPP167. We observed the most severe changes among households without health insurance, which registered a -26.4% (95% CI = -30.8, -22.0) drop in THE, -18.9% (95% CI = -21.9, -15.8) for FE, and -32.4% (95% CI = -37.7, -27.1) for CTP, as well as increases of 22.8% (95% CI = 17.7, 28.0) in the prevalence of positive HE and 33.5% (95% CI = 21.3, 45.7) in the HE/CTP ratio (**Table 2**).

**Table 2:** Health expenditure in households according to health insurance status, Mexico 2018 and 2020*

					If reported HE>0	
	**THE, US$**	**FE, US$**	**CTP, US$**	**Reported HE>0, %**	**HE, US$**	**HE out-off CTP, %**
**Overall (2018-2020)**						
2018	3162.0 (3115.6, 3208.5)	1164.4 (1152.2, 1176.7)	2262.8 (2217.9, 2307.7)	62.7 (61.9, 63.4)	180.0 (172.0, 188.0)	7.394 (7.2, 7.6)
2020	2815.0 (2782.6, 2847.4)	1041.5 (1,032.3, 1050.6)	1979.5 (1948.7, 2010.4)	73.4 (72.8, 74.0)	166.6 (160.6, 172.5)	7.391 (7.2, 7.5)
*Relative change*	-11.0 (-12.6, -9.3)	-10.6 (-11.8, -9.3)	-12.5 (-14.7, -10.3)	17.2 (15.5, 18.9)	-7.5 (-12.7, -2.2)	-0.035 (-3.14, 3.07)
**According to health insurance – nothing**						
2018	3345.6 (3171.2, 3519.9)	1167.1 (1130.3, 1203.9)	2536.0 (2366.3, 2705.6)	54.8 (52.8, 56.9)	133.8 (111.5, 156.1)	5.3 (4.9, 5.6)
2020	2463.4 (2390.9, 2535.9)	947.0 (927.6, 966.3)	1714.8 (1645.0, 1784.6)	67.3 (66.0, 68.6)	137.4 (122.8, 152.0)	7.0 (6.6, 7.4)
*Relative change*	-26.4 (-30.8, -22.0)	-18.9 (-21.9, -15.8)	-32.4 (-37.7, -27.1)	22.8 (17.7, 28.0)	2.7 (-17.6, 22.9)	33.5 (21.3, 45.7)
***Seguro Popular* and/or INSABI†**						
2018	2050.0 (2,018.5, 2081.6)	920.3 (907.7, 932.8)	1237.2 (1210.4, 1263.9)	63.5 (62.4, 64.6)	134.0 (125.3, 142.7)	8.6 (8.3, 8.8)
2020	1920.2 (1,890.7, 1949.6)	866.0 (854.6, 877.5)	1149.7 (1124.8, 1174.7)	73.3 (72.3, 74.3)	124.6 (115.9, 133.3)	8.2 (8.0, 8.5)
*Relative change (%)*	-6.3 (-8.4, -4.3)	-5.9 (-7.7, -4.1)	-7.1 (-9.9, -4.2)	15.4 (12.9, 18.0)	-7.0 (-15.9, 1.8)	-3.7 (-8.2, 0.7)
**Social security**						
2018	4019.2 (3941.0, 4097.4)	1331.6 (1310.7, 1352.5)	3089.7 (3013.4, 3165.9)	61.6 (60.5, 62.6)	217.8 (203.2, 232.4)	6.7 (6.4, 7.0)
2020	3361.9 (3314.1, 3409.8)	1141.2 (1127.6, 1154.9)	2498.3 (2452.0, 2544.5)	74.2 (73.4, 75.0)	194.9 (185.0, 204.8)	7.0 (6.8, 7.2)
*Relative change (%)*	-16.4 (-18.4, -14.3)	-14.3 (-16.0, -12.6)	-19.1 (-21.6, -16.7)	20.5 (18.1, 22.9)	-10.5 (-18.1, -3.0)	4.0 (-1.3, 9.4)
**Mixture or private**						
2018	3070.1 (3010.9, 3129.3)	1202.9 (1183.7, 1222.1)	2060.0 (2004.8, 2115.2)	66.9 (65.7, 68.2)	193.7 (173.3, 214.1)	7.7 (7.3, 8.1)
2020	2764.1 (2717.4, 2810.9)	1085.4 (1070.5, 1100.4)	1824.0 (1781.1, 1867.0)	78.0 (76.9, 79.1)	166.0 (154.8, 177.2)	7.8 (7.5, 8.2)
*Relative change*	-10.0 (-12.3, -7.7)	-9.8 (-11.7, -7.9)	-11.5 (-14.6, -8.3)	16.6 (13.9, 19.3)	-14.3 (-25.0, -3.6)	1.5 (-5.1, 8.1)

THE – total quarterly household expenditure, FE – total quarterly food expenditure, CTP – total quarterly capacity to pay, HE – total quarterly health expenditure, INSABI – Instituto de Salud para el Bienestar

*Data presented as calculated average or percentage (95% CI) unless specified otherwise.

†Data from 2018 and 2020 waves of the National Household Income and Expenditure Survey (ENIGH). All relative changes and 95% CI were computed through nonlinear combination of post estimated parameters based on the delta method, using *nlcom* command of the Stata statistical package used.

**Figure 1:**
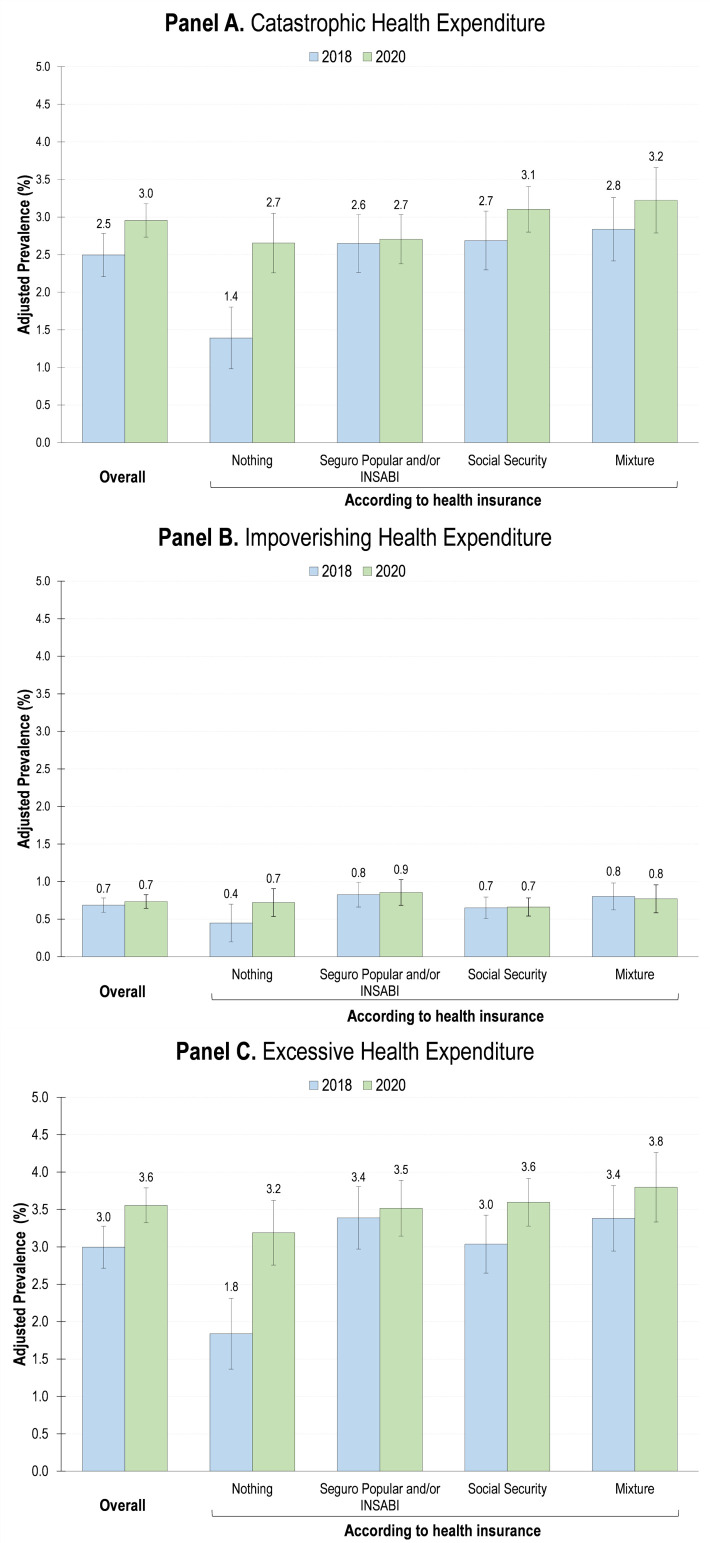
Adjusted prevalence of catastrophic, impoverishing, and excessive health expenditure by health insurance status, Mexico, 2018-2020. Data from 2018 and 2020 waves of the National Household Income and Expenditure Survey (ENIGH). Probabilities of CHE, IHE and EHE were estimated adjusting a two-stage probit or Heckprobit model [48]. We adjusted the first stage for all covariates described above, for the interaction between survey year and health insurance, and by state and survey year fixed effects; meanwhile, we adjusted the selection equation for all control variables mentioned above (except for the household composition, the proportion of family members aged 0-5 or ≥65 years and with a disability), and for state and survey year fixed effects.

Regression models’ results show that the overall prevalence of CHE and EHE increased by 18.4% (95% CI = 6.1, 30.7), and 18.7% (95% CI = 7.9, 29.5), respectively (**Figure 1**, Panels A and C). Of the total number of households with health needs generating EHE, 15.6% registered IHE, 11.7% both CHE and IHE, and 72.7% CHE. We only observed significant increases in the probability of CHE, IHE, and EHE among households without health insurance (CHE: 90.7%; 95% CI = 31.6, 149.7 and EHE: 73.5%; 95% CI = 25.3, 121.8) (**Figure 1**). The results remained consistent in the sensitivity analyses (Appendix 1 in the **Online Supplementary Document**).

**Figure 2:**
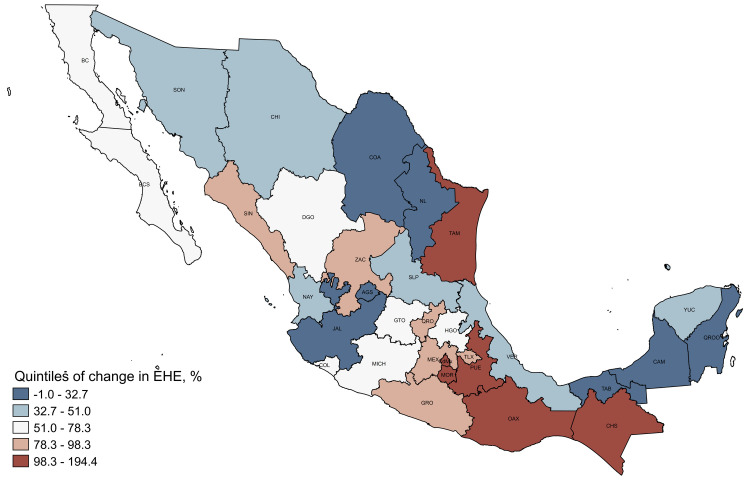
Quintiles of changes in adjusted prevalence of excessive health expenditure by state, Mexico, 2018-2020. Data from 2018 and 2020 waves of the National Household Income and Expenditure Survey (ENIGH). We estimated probabilities of CHE, IHE, and EHE by adjusting a two-stage probit or Heckprobit model [48]. We adjusted the first stage for all covariates described above, for the interaction between survey year and health insurance, and for state and survey year fixed effects; meanwhile, we adjusted the selection equation for all control variables mentioned above (except for the household composition, the proportion of family members aged 0-5 or ≥65 years and with a disability) and for state and survey year fixed effects. We computed percentage changes between 2018-2020 through nonlinear combination of post estimated parameters based on the delta method, using *nlcom* command of Stata package.

The increase in the proportion of households without health insurance incurring in EHE was distributed unevenly across the territory (range = -1.0%, -194.4%) (**Figure 2**). Virtually all Mexican states (n/N = 31/32) recorded an increase in EHE among households without health insurance (except Aguascalientes, which recorded a one percent reduction). Central states such as Mexico, Mexico City, Morelos, Puebla and Guerrero, and Southern states such as Oaxaca, and Chiapas (the last three being the poorest in the country) registered the highest increases. In contrast, the northern, riches states registered much more modest increases in EHE among households with no health insurance.

## DISCUSSION

The exclusion of population groups from financial protection schemes, such as public health insurance, increases catastrophic spending and poverty [8]. For that reason, one of the main objectives of *Seguro Popular* was to provide financial protection to the uninsured population (workers with informal jobs, the self-employed, and the unemployed) [26]. CHE and IHE were affecting a large proportion of the Mexican households at the turn of the century, especially those without social security. Several studies showed that *Seguro Popular* was successful in protecting households from CHE and IHE [26,29,33,50,51]. Despite these achievements, the Mexican government decided to substitute it with *INSABI* in early 2020 based on the emergence of corruption scandals and substantiated demands for improved coverage, effectiveness, and quality.

The impact on health care coverage of this policy decision was first documented by the National Council for the Evaluation of Social Policies. According to its report on the Multidimensional Measurement of Poverty in Mexico, the ‘’deprivation due to access to health care services” (a concept used to describe the lack of access to health care) increased by 75% in the first two years of the new federal administration, from 16% of the total population (20.1 million people) in 2018 to 28% (35.7 million) in 2020 [52].

The decline of health care coverage, in turn, was associated to an expansion in the use of private health care services and health care expenditure. A high use of private services has been observed at least since 2012, and it has been higher in the population without health insurance. According to the Health and Nutrition Survey (*ENSANUT*) data, 66% of the population reporting a health problem used private services in 2012, 59% in 2018, and 60% in 2021 [53]. Additionally, the ENIGH 2018 and 2020 shows that the average household expenditure in health in Mexico grew from 901 constant pesos in 2018 to 1266 pesos in 2020, a 40% increase [54]. Part of this increase could also be attributed to the growth of the demand of private health services associated to the COVID-19 pandemic.

According to our data, the number of households without health insurance increased from 3 million in 2019 to 5.8 million in 2020, a figure not seen in Mexico since the early 2000s, during the initial years of implementation of the *Seguro Popular* reform. Indigenous, poor, and female-headed households and those with low schooling levels and with senior individuals were the most affected. This means that the decline in health care coverage affected mostly vulnerable households and those in central and southern states. This finding is consistent with the fact that a very large proportion of the indigenous population and a large proportion of the population in the poor, central, and southern states lack social security and receive health care from the agencies tending the uninsured, the ones mostly affected by the policy changes implemented between 2018 and 2020.

The decline in health care coverage, in turn, was associated to a 20% increase in the prevalence of EHE, reaching levels not seen in Mexico since 2004-2006 [36]. The increase in the prevalence of EHE was higher in households without health insurance and those in the poorer, southern states of the country, where a large proportion of the population lacks social security. While we cannot establish causality based on this data, part of the increase in EHE is likely due to the dismantling of *Seguro Popular*. Further studies using more granular and administrative data are needed to estimate the specific effect of this policy change on EHE.

The results of this study have some limitations. First, although the estimated models considered an exhaustive set of predictors for the outcomes of interest [29,37-43], this analysis is subject to the limitations of any observational study (i.e. biases due to omitted variables). For example, the data did not allow us to identify the specific type of health problem that caused the out-of-pocket expenses or the household member whose health care generated the costs. In this sense, and even though it is an accepted practice, defining households as the unit of analysis does not allow us to consider family diversity. Second, although the analysed survey is considered the gold standard for measuring spending in Mexican households, we cannot discard possible recall biases in self-reported spending, specifically, health-related expenditure. Finally, while we did not estimate a direct causal effect of the dismantling of *Seguro Popular* in the increase of EHE, we argue that the increase in EHE between 2018 and 2020 is probably due to the combined effect of four events: a 20% decrease in the budget of the Ministry of Health (MoH) in the period 2015-2019 (which affected the supply of health care services for the uninsured population) [55], the dismantling of *Seguro Popular* (which reduced public health care coverage and stimulated the use of private health services), the cancellation of CCT programs such as *Prospera* (which reduced the capacity to pay of poor, uninsured households) [56], and the COVID-19 pandemic (which also increased out-of-pocket expenditure through the use of private health services, given that an important share of public facilities were focused on caring for COVID-19 patients).

Our results do not allow us to distinguish the specific effect of these four events. However, a chronology issue suggests that the policy changes were largely responsible for the increasing prevalence of EHE. Some policy changes (reduction of the MoH budget) started to manifest their effect since 2015, which was strengthened in 2019 with the cancellation of *Prospera* and the dismantling of *Seguro Popular*. In contrast, the COVID-19 pandemic started to exhibit its effect by mid-2020. The first cases of COVID-19 in Mexico were registered at the end of February of 2020 and the first peaks of the epidemic occurred in the middle of that year [57]. The ENIGH, in turn, was implemented from 21 August to 28 November 2020, and measured spending in the previous quarter. This means that the survey captured the multi-annual effects of the policy changes but only the initial effects of the pandemic. This reinforces the plausibility of the hypothesis that the increase in EHE was mostly due to the decrease in the budget of the MoH in the period 2015-2019, the dismantling of *Seguro Popular* and the cancellation of the CCT programs.

Future studies should estimate the increase in out-of-pocket expenditures in health and EHE attributable to the reduction of health care coverage due to the substitution of *Seguro Popular* with *INSABI*, and to the COVID-19 pandemic. The study of the evolution of EHE in the post-pandemic period (in the absence of one of these two variable) will help determine the specific effects of the recent policy changes implemented in Mexico.

## CONCLUSIONS

Our four main findings are as follows:

1. The discontinuation of *Seguro Popular* and its substitution for INSABI had negative effects on health care coverage and was associated to an increase in the prevalence of EHE.
2. The reduction in health insurance coverage was more severe in female-headed households, those with lower schooling, those with lower participation in the labour market, those with indigenous families, and those living below the poverty line.
3. The increase in the prevalence of EHE affected vulnerable households mostly, and, given that they were living in poverty, this impact is likely to have been especially devastating on living conditions.
4. There was a large variability across states in the changes in financial protection which is in part explained by the huge difference in access to social security between northern and southern states.

We call for a need to strengthen institutions in Mexico and other low- and middle-income countries with fragmented health systems to guarantee the right to the financial protection in health. This right should prevail over any normative, operational, or fiscal arrangements associated to a specific government.

## Additional material


Online Supplementary Document
