# Supplementary material for: Increase of catastrophic and impoverishing health expenditures in Mexico associated to policy changes and the COVID-19 pandemic
Source: J Glob Health. 2023 Oct 27;13:06044. doi: 10.7189/jogh.13.06044 (PMC10602209; doi:10.7189/jogh.13.06044)
Supplement: Online Supplementary Document [file jogh-13-06044-s001.pdf]

Online Appendix 1. **Sensitivity analysis for the adjusted prevalence of CHE and EHE by health insurance status, Mexico, 2018-2020[a]**

	Threshold: HE/CTP ≥25%		Threshold: HE/CTP ≥40%	
	CHE	EHE	CHE	EHE
	Estimated percentage (95% CI)			
Overall (2018-2020)				
2018	3.6 (2.9, 4.4)	4.1 (3.5, 4.7)	1.7 (1.1, 2.3)	2.3 (1.9, 2.7)
2020	4.3 (3.7, 4.9)	4.8 (4.3, 5.3)	1.9 (1.5, 2.4)	2.6 (2.3, 3.0)
Relative change (%)	*17.3 (4.2, 30.3)*	*17.3 (6.5, 28.0)*	*15.0 (-5.9, 35.9)*	*14.7 (0.6, 28.7)*
According to health insurance				
Nothing				
2018	2.2 (1.5, 3.0)	2.7 (1.9, 3.4)	0.8 (0.3, 1.2)	1.3 (0.8, 1.7)
2020	4.0 (3.2, 4.8)	4.4 (3.7, 5.2)	2.0 (1.4, 2.7)	2.7 (2.1, 3.3)
Relative change (%)	*78.4 (28.4, 128.4)*	*65.6 (23.8, 107.4)*	*161.2 (46.4, 276.1)*	*115.5 (38.7, 192.4)*
Seguro Popular and/or INSABI[b]				
2018	3.9 (3.0, 4.8)	4.6 (3.9, 5.4)	1.8 (1.1, 2.5)	2.7 (2.2, 3.3)
2020	4.1 (3.4, 4.7)	4.8 (4.2, 5.4)	1.8 (1.4, 2.3)	2.7 (2.3, 3.1)
Relative change (%)	*4.1 (-11.9, 20.1)*	*4.0 (-9.9, 17.9)*	*-0.9 (-25.1, 23.2)*	*-0.8 (-18.1, 16.4)*
Social Security				
2018	3.9 (3.0, 4.7)	4.2 (3.5, 4.8)	1.9 (1.2, 2.6)	2.3 (1.8, 2.8)
2020	4.4 (3.7, 5.0)	4.8 (4.3, 5.4)	2.0 (1.5, 2.5)	2.5 (2.1, 2.9)
Relative change (%)	*13.4 (-2.6, 29.4)*	*16.1 (1.4, 30.8)*	*4.7 (-19.0, 28.3)*	*8.5 (-10.6, 27.6)*
Mixture or private				
2018	4.0 (3.2, 4.8)	4.5 (3.8, 5.2)	1.9 (1.3, 2.6)	2.6 (2.1, 3.1)
2020	4.6 (3.9, 5.3)	5.1 (4.4, 5.8)	2.0 (1.5, 2.5)	2.7 (2.2, 3.2)
Relative change (%)	*14.0 (-5.7, 33.7)*	*13.4 (-4.5, 31.3)*	*3.5 (-22.7, 29.7)*	*5.8 (-15.2, 26.9)*

[a] Data from 2018 and 2020 waves of the National Household Income and Expenditure Survey (ENIGH). Probabilities of CHE, IHE and EHE were estimated adjusting a two-stage probit or Heckprobit model[48]. The first stage was adjusted by all covariates described above and by the interaction between survey year and health insurance, and by state and survey year fixed effects; meanwhile, the selection equation was adjusted by all control variables mentioned above (except by the household composition, the proportion of family members aged 0-5 or ≥65yrs and with a disability) and by state and survey year fixed effects. All relative changes and 95% CI were computed through nonlinear combination of post estimated parameters based on the delta method, using *nlcom* command of Stata package.
